# A Mobile App (CareFit) Supporting Physical Activity for Informal Carers of People With Dementia: Mixed Methods Feasibility and Adaptation Study

**DOI:** 10.2196/56739

**Published:** 2025-08-29

**Authors:** Kieren J Egan, William Hodgson, Bradley Macdonald, Ramsay Meiklem, Ryan Innes, Alison Kirk, Barbara Fawcett, Mark D Dunlop, Roma Maguire, Greg Flynn, Joshua Stott, Gill Windle

**Affiliations:** 1 Department of Computer and Information Sciences Digital Health and Wellness Research Group (DHAWG) University of Strathclyde Glasgow United Kingdom; 2 Physical Activity for Health Research Group University of Strathclyde Glasgow United Kingdom; 3 Department of Social Work and Social Policy University of Strathclyde Glasgow United Kingdom; 4 School of Health Sciences Bangor University Bangor United Kingdom; 5 ADAPTlab Department of Clinical, Educational and Health Psychology University College London (UCL) London United Kingdom

**Keywords:** physical activity, Android, Apple, intervention, co-design, exercise, app, development, support, carer, dementia

## Abstract

**Background:**

Informal carers are critical in looking after people with dementia. The value of all informal care as a total in the United Kingdom is now estimated to be £184 billion (US $249 billion). However, many carer groups are inadequately supported and face poor mental and physical health with limited opportunities for physical activity. There is a public health priority to address such challenges and a lack of innovation regarding approaches to promote physical activity.

**Objective:**

This study aimed to co-design, adapt, and explore the feasibility of a novel cross-platform approach to support physical activity among carers of people with dementia.

**Methods:**

Project stages included a co-design phase followed by a feasibility study guided by both the reach, effectiveness, adoption, implementation, and maintenance and Medical Research Council complex intervention frameworks. Co-design involved 3 development “sprints,” gaining feedback from a range of stakeholders (eg, carers, support professionals, charities, researchers, and developers) and identifying priority areas. Our feasibility study involved the evaluation of our recruitment, intervention, and outcome approaches over 8 weeks (target population: N=50) with participants. Participants were recruited from local community networks in Scotland as well as research study registries.

**Results:**

We successfully co-designed, developed, and user tested the CareFit app. Final app design included a simplified navigation system and increased delivery of video content as well as a more personalized delivery approach. In total, 41 carers of people with dementia were recruited, with 21 (51%) completing the 8-week study. Study retention was considerably lower for carers with higher levels of physical activity at baseline as opposed to those with lower levels (5/14, 36% vs 16/27, 59%, respectively). CareFit was rated as “acceptable” on the System Usability Scale, and we observed common user patterns of behavior (eg, an initial focus on the Learn section). The feasibility study results demonstrated that the intervention appeared safe for use (no adverse events reported) and the video content for carers was well received. A greater depth of social elements appears critical for future development. Although professional stakeholders did not reach consensus on the outcome of greatest utility, the outcome measures tested were largely suitable for future use in this group, including novel sedentary behavior and muscle and balance measures.

**Conclusions:**

Many carers of people with dementia do not have the same access to physical activity as noncarers. Our findings show key challenges regarding recruitment and retention. Although we cannot currently recommend progression to a randomized controlled trial, we conclude that further work is needed to better understand the “active ingredients” of the intervention outlined. This includes exploring the delivery of a preventative intervention earlier in the carer trajectory—something critical for adoption and long-term use.

**International Registered Report Identifier (IRRID):**

RR2-10.2196/53727

## Introduction

Health and social care models worldwide are facing perpetual crises. Aging populations, increasing comorbidities, and a shrinking health and care workforce create a “perfect storm” of continuous system decline [1]. Caring roles are increasingly taken on by the community, often by informal (family) carers [2]. In the United Kingdom, 10 million informal carers save the medical system £184 billion (US $249 billion) each year; the cost of a second National Health Service (NHS) [3]. NHS data reveal that carer-reported quality of life is decreasing year upon year, and recent national-level surveys in the United Kingdom (engaging >10,000 carers) indicate that 82% of carers see challenging impacts of delivering care on their physical and mental health [4-6]. There is a compelling need to recognize this paradigm shift and build much greater resilience in our care system and networks. This includes asking difficult implementation questions as to how scalable solutions for carers can be co-designed, implemented, and maintained.

Across a growing array of technology [7,8], the development of digital supports for carers of people with dementia continues to be of significant societal interest [9-11]. UK data suggest that 1 in 2 people will be affected by dementia in their lifetime—either by the condition or by caring for someone with dementia (944,000 people currently live with dementia in the United Kingdom) [12]. Understandably, dementia has long been a topic of interest for developing digital interventions, including the pioneering iSupport platform developed by the World Health Organization that has facilitated evidence-based theory such as personhood and cognitive reframing [13,14] becoming accessible to a global audience through online training and support. While iSupport delivers key material of critical importance (eg, introducing dementia, dementia-related behaviors, and carer health and well-being), there remains scope to increase the capacity of the platform to provide personalized advice to support the regular physical activity of carers. More broadly, physical activity among carers is a surprisingly underdeveloped area of current science. Multiple reviews have highlighted that carers are largely overlooked when it comes to physical activity innovations (of both dyadic and independent carer use), with systematic reviews identifying between 11 and 14 interventions to date aimed at carers, including a strong emphasis on face-to-face and telephone delivery [15,16]. Given this gap in knowledge, a team of multidisciplinary researchers at the University of Strathclyde co-designed and evaluated (with carers and health professionals) a prototype digital tool, CareFit, aimed at increasing physical activity (and reducing sedentary behavior) in carers [9]. Awareness of physical activity guidelines (eg, from the World Health Organization and the UK government [17]) can be poor among vulnerable groups [10], including carers [18]. Therefore, the platform was built with an emphasis on “actionable information,” particularly for those starting out in physical activity, and our original work was conducted during the COVID-19 pandemic lockdowns. The intervention design was largely influenced by the transtheoretical model of behavior change [11,19], is self-led by informal carers at their own pace, and does not require specialist equipment. The initial (3-week) co-design and pilot study across Scotland [18,20] successfully demonstrated the initial acceptability, feasibility, and usability of the approach, which was open to use across any carer group. Remaining gaps in knowledge include a more in-depth understanding of the practicalities of delivering CareFit within a single postdiagnostic pathway across recruitment, adherence, and outcomes. This includes critical questions regarding how to personalize the intervention, how to extend the platform beyond 3 weeks of use, and the identification of key barriers and enablers to facilitate more widespread and scalable delivery. We also sought to simplify the use of CareFit from previous studies and consider more human and community elements—particularly given key challenges regarding the digital divide [20] for informal carers. Thus, we set out to co-design, adapt, and explore the feasibility of a novel cross-platform approach to support physical activity in carers of people with dementia.

## Methods

### Objectives

Our aim was to co-design, adapt, and personalize a cross-platform digital health app (CareFit) designed to support regular physical activity in carers of people with dementia for a period of 8 weeks and evaluate the potential for implementation.

Our objectives were to (1) expand an initial 3-week intervention to an 8-week intervention to support maintenance of physical activity; (2) develop an understanding of recruitment pathways and explore barriers to and enablers of recruitment for a future definitive trial, including recruiting from more deprived socioeconomic groups; (3) improve the understanding of use adherence and develop an understanding of the most reliable methods to regularly measure physical activity and sedentary behavior among dementia carers; (4) explore how CareFit could provide added value for dementia carers with other existing digital interventions (such as iSupport) through qualitative interviews or focus groups with key stakeholders; and (5) explore unexpected benefits, including whether people with dementia also see the benefits of CareFit.

### Study Design

#### Overview

The CareFit for dementia carers study was a concurrent mixed methods [21] evaluation of a novel motivational mobile app to support home-based regular physical activity for informal dementia carers. The study methodology has been published previously [22] and forms a stand-alone element of an existing randomized controlled trial of iSupport [23]. The mixed methods evaluation was informed by both the Medical Research Council (MRC) complex intervention framework [24-26] and the reach, effectiveness, adoption, implementation, and maintenance (RE-AIM) framework [27].

For initial co-design to achieve both adaptation and expansion, we used 3 development “sprints” where different elements of the app were sequentially developed with our local development partner, Add Jam [28]. Feedback was sought from a range of stakeholders such as carers and professionals as well as researchers and developers to create priority areas for app development. We were also able to build on priorities from previous work on CareFit and the feedback obtained [24]. Our subsequent feasibility study involved both local and national networks in Scotland as well as the use of Join Dementia Research (JDR) to understand the feasibility of recruiting for a larger trial. We aimed to recruit 50 carers of people with dementia for our feasibility study. This number was arrived at based on both related statistical guidance for pilot studies and anticipated high dropout [14,29].

#### Feasibility Study Inclusion Criteria for Carers

The inclusion criteria for carers were as follows: (1) adults (aged ≥18 years) living in Scotland who self-identified as informal carers (eg, partners, children, or friends) of a person with dementia (self-reported), (2) contemplating or preparing to practice physical activity, (3) ability to carry out simple exercises such as arm raises or stretching, (3) ability to read and write in English, (4) access to a smartphone (Android or Apple) as well as access to the internet, and (5) normal or corrected-to-normal eyesight.

#### Feasibility Study Exclusion Criteria for Carers

The exclusion criteria for carers were as follows: (1) anyone advised by a clinician not to undertake physical activity or make any change in their present level of exercise, (2) already regularly exercising to a significant level outside the home (eg, running or cycling), (3) residence outside Scotland at the time the study was conducted, and (4) current participation in a related iSupport study.

#### Feasibility Study Inclusion Criteria for Professionals

The inclusion criteria for professionals were as follows: (1) adults (aged ≥18 years) living in Scotland and working as health and social care professionals and (2) willingness to engage with the study (eg, share information about CareFit with carers of people with dementia) through their professional roles for a period of at least 3 months (to ensure timely follow-up).

#### Feasibility Study Exclusion Criteria for Professionals

The exclusion criteria for professionals were work exclusively within the NHS.

### Ethical Considerations

Ethics approval for feasibility work was obtained through the Bangor University School of Medical and Health Sciences Academic Ethics Committee (approval 2021-16915). The initial co-design and adaptation work was supported through approval 2022 from the University of Strathclyde, Department of Computer and Information Sciences. CareFit was coded using React Native (Meta Platforms) so that we could facilitate use across both Android and Apple mobile phones and tablet devices. As the study was a “closed” research project (ie, not available freely to the public), onboarding of participants involved a number of steps regarding contacting the research team, confirming that the participants met the study inclusion criteria, assigning participants a study ID, and then sending them a bespoke link for the corresponding Apple and Google stores. This process was thoroughly tested before the implementation study began. The protocol publication provides further information [22]. Informed consent was obtained from participants in the studies after reading a participant information sheet and given an opportunity to ask questions. Participants were given a small thank you token for taking part in a study. All sessions will be conducted in a way to ensure privacy and confidentiality.

### Research Team and Reflexivity

The research team conducting qualitative interviews comprised 3 (male) research assistants (WH, RM, and RI), who have expertise undertaking qualitative work within digital health research, and the project lead (male; KJE), who has >10 years of postdoctoral experience and a specialist interest in carer populations. The interviews and focus groups were facilitated by all 4 team members (WH, RM, RI, and KJE)

### Data Collection, Management, and App Download Process

**Figure 1:**
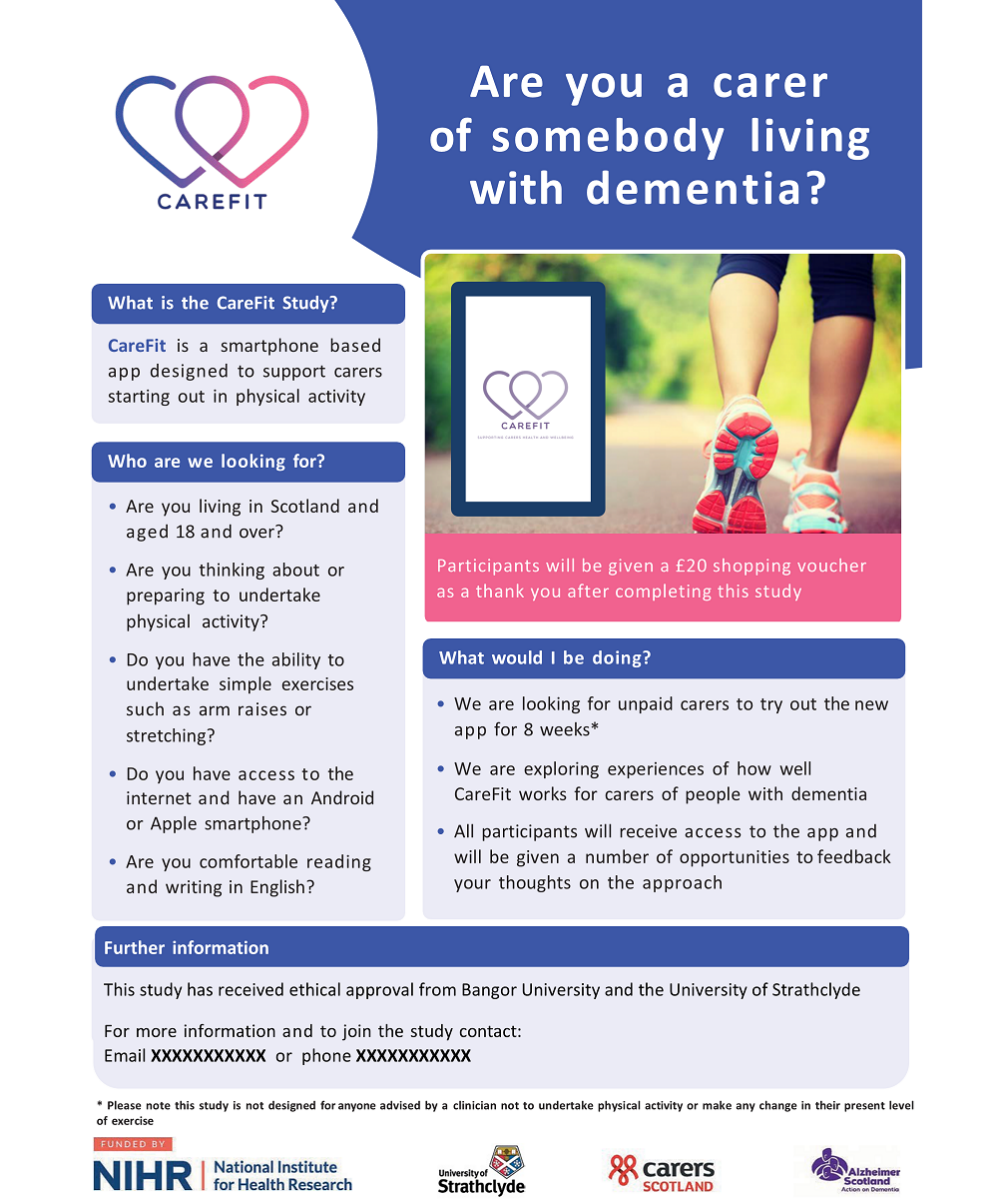
Recruitment flyer for carer participants.

Study advertisements (in both paper and digital formats) were shared with study partners in parallel with the use of the JDR platform (Figure 1). Beyond monitoring study interest numbers, our key areas of data collection included (1) online surveys, (2) “in-app” data collection, and (3) interviews and focus groups. For online surveys, information was shared using the online platform Qualtrics (Qualtrics International Inc) [30]. Carers who consented were directed to a bespoke hyperlink within the Google Play Store and Apple App Store where an individual pseudoanonymized ID was entered after download. For in-app data collection, data included use and time stamps for users across the app tabs, such as Activity, Planner, Resources, and Sharing.

### Qualitative Data Collection

For an overview of the qualitative approach, Multimedia Appendix 1 provides the full COREQ (Consolidated Criteria for Reporting Qualitative Research) checklist [31]. The research assistants (WH, RM, and RI) had previous background experience in qualitative work within digital health research across a wide range of chronic conditions (eg, cancer, diabetes, and kidney failure). All staff members had conducted qualitative studies previously, including at the master’s in research (WH and RI), PhD (RM), and postdoctoral (KJE) levels, where training was supported by fellow staff members and university training courses. The researchers had no professional or ongoing relationship with informal carer participants, and the latter were recruited through convenience sampling—however, a small number of the professionals interviewed at the conclusion of the work were identified through university roles (n=2). All participants were informed that researchers were interested in exploring the use of digital approaches to support health and wellness for carers specifically through physical activity. We repeatedly shared with participants that there were no “right” answers and that relationships or care would not be affected in any way by the responses given. We asked all participants for basic demographic information so that we could accurately describe our study samples.

Interviews and focus groups were conducted online, and the format was internally pilot-tested before use. There were no nonparticipants present in any of the interviews, and no repeat interviews were conducted. The interviews lasted approximately 30 to 45 minutes, and we did not capture field notes for the focus groups. Audio recordings were checked against transcripts to ensure accuracy, but the transcripts were not sent back to participants for checking. Each researcher coded their own individual interviews, which were then cross-checked and discussed with another member of the team. Although we approached individuals who left the study for feedback as to why they dropped out, we did not manage to obtain such feedback.

### Adaptation of CareFit

**Figure 2:**
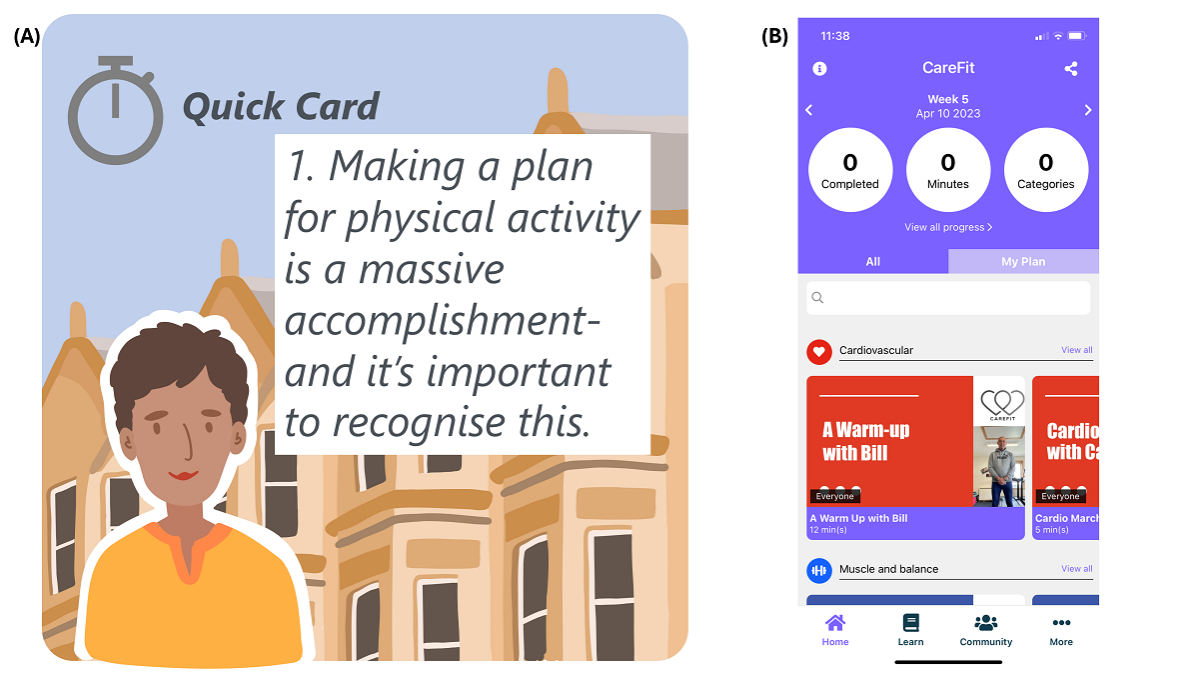
(A) Personas developed for the CareFit app used throughout the Learn section and (B) the Home tab explaining pictorially how the user interface was designed.

We successfully expanded and adapted CareFit for 8 weeks of use within the context of dementia care (Table 1 and Figure 2). This included developing features highlighted through previous real-world use of a first-edition app [18,20], including aspects such as a redesign or simplification of the graphical user interface. We also redesigned the physical activity planner (placed on the Home tab) so that users could both add and review activities using the same screen and add their own activities. During our 3 development sprints, we presented progress across a range of charity partners (including Alzheimer Scotland) and also invited stakeholder partners to try out elements of the app by downloading prototypes in development. Co-design outputs (Table 1) placed a strong emphasis on carer feedback from 16 carers who had previously used a version of the app for 3 weeks and told us priorities for improvement. This feedback was integrated with charity partner feedback and technical feasibility feedback to adapt the requirements accordingly. Simplification ultimately led to 4 tabs: Home, Learn, Community, and More. Following recommendations for simplification, we established a uniform image set across all activity videos and color coded these according to whether the activity was cardiovascular (red), sedentary (gold), or related to muscle and balance (blue), reflecting UK physical activity guidelines. Additional elements included short video clip animations summarizing key information across the Learn section; the development of fictional personas that described a variety of dementia caring scenarios across different genders, geographical settings, and ages; and links to local resources within the Community tab.

**Table 1:** Overview of the components developed for CareFit, including the Home, Planner, Learn, Community, and More sections as well as the content management system (CMS).

Section	Purpose	Implementation and personalization detail
Home—Activities section	To allow participants to easily access all app functions, including the exercise videos and weekly progress planner; review their progress; and plan ahead	We developed a breadth and depth of video content, including 10 cardiovascular, 10 muscle and balance, 4 breaks from sedentary time, and 2 mindfulness videos as well as 2 mindfulness audios, including videos of up to 10 min. All videos developed were hosted on a “hidden” YouTube channel that enabled full captioning for those who are deaf or hard of hearing.
Home—Planner section	To allow users to develop their physical activity plans at a range of starting points	Users were provided with 3 simple statistics on their progress—the number of tasks completed, the number of min of physical activity, and the number of different categories of physical activity carried out. Users were provided with additional information on the number of activities and min planned with a comparison of how many min were carried out. We designed the planner so that participants could build a weekly “rolling” routine and habits could be built up over time. Adding carers’ own activities (eg, going for a walk) was a functionality that we also added to the planner in a prospective manner. A green ring fills up around this according to the percentage they have or have not met.
Learn	Introduce physical activity for those contemplating increasing their current levels	There were 7 stages developed in total. Each “stage” was designed to take no more than 10 min. We developed both fictional personas of carers across Scotland in collaboration with Carers Scotland and a graphic illustrator to represent different ages, genders, and ethnicities, as well as to represent different geographical locations. In total, 6 different backgrounds were developed with 6 different people to represent carers (eg, different ages, ethnicities, and genders). We developed a series of summary “quick cards” as well as video summaries of key points and the purpose of different sections (Figure 2). We developed novel videos for guided meditation to support carers, including working with a specialist who works with carers on a regular basis. This resulted in 2 carer meditation videos and 2 carer audio meditations.
Community	Provides external links to outside agencies, stakeholders, support groups, and physical activity projects such as Paths for All	We developed a list of 25 local resource links within the app across the domains of physical activity, mental health, and carer support. For example, specific community links (including hyperlinks that give direct access to websites) included NHS^a^ physical activity and mindfulness material and physical activity support groups such as Couch to 5K, Cycling Scotland, Paths for All, and walking groups. There was also a functionality built in to create user stories so that individual carers or organizations could share updates on app use as the user base grows.
More	To give users the ability to update the app and delete data	The More section allowed for a variety of additional functions as a quick list, including making it easier for users to change alarm permissions for activities and delete data, as well as an area for seeing updates or learning more about the research team.
CMS	In addition to the aforementioned elements was the development of a bespoke CMS that allows the research team to upload materials to the app schema in real time according to the project needs	For example, this means that all the images shown in Figures 2-4 can be added, deleted, or modified by the research team whenever this is required. The system also facilitated the collection of user data so that we could explore individual patterns of use of carers using the app.

^a^NHS: National Health Service.

### Evaluation Outcomes

Our mixed methods evaluation was designed to improve our foundational understanding of CareFit supported by validated instruments wherever possible. This included measures to understand usability (System Usability Scale [SUS] [32], which is a widely used 10-item Likert scale to provide a global view of subjective assessments of usability), improvements in quality of life and economic impact (EQ-5D-5L [33], which is a 5-point scale covering domains of mobility, self-care, usual activities, pain or discomfort, and anxiety or depression), and changes in physical activity or sedentary behavior (International Physical Activity Questionnaire–Short Form [IPAQ-SF] [34]). The IPAQ-SF has been developed and tested for use in adults (aged 15-69 years) and asks individuals to report the number of days and time spent performing moderate and vigorous physical activity, walking, and sitting. We sought to deepen our contextual understanding of the population by capturing information on demographics (eg, that could be used as covariates in later analyses) through baseline and follow-up questions on age group, gender, and number of years of caring and hours of caring per week. Specific questions for informal carers also included information to allow for the calculation of BMI, a single question on sedentary behavior and muscular activity, and familiarity with and awareness of physical activity. Professional and carer interviews and surveys were structured around the RE-AIM and MRC complex intervention frameworks (Multimedia Appendices 2 and 3). Other data collected included in-app data, which primarily supported our understanding of intervention adherence (and dropout).

### Analysis

Our use of RE-AIM focused on the planning and conduct of data gathering as opposed to the evaluation phases (see the Limitations section). To better understand uncertainty regarding key elements of the MRC complex intervention framework, we present key results structured around recruitment, intervention design, and suitability of outcomes. Our approach to mixed methods [21] was to analyze qualitative and quantitative results individually in parallel and combine them to draw conclusions. The interviews were recorded using an encrypted dictaphone and university-approved video software.

Thereafter, the thematic analysis process by Braun and Clarke [35] was followed using NVivo (version 12; Lumivero) and was guided by interpretivism and the individual perspectives that informal carers and professionals can provide. We chose thematic analysis due to particular strengths regarding exploring perceptions, attitudes, and motivations and finding deeper meanings as opposed to the more rigid framework of content analysis. Due to the diverse nature of the population interviewed, we did not anticipate reaching data saturation but, instead, invited as many professionals and informal carers to come forward as possible (through convenience sampling) within the time limits of the work. We did not conduct a formal coding tree but discussed notes and reflections together as a group. Deductive approaches were supported primarily through the use of the complex intervention framework (namely, feasibility of recruitment, intervention design, and outcome suitability). The minor subthemes presented were derived from the data throughout, and examples of quotes are provided consistently.

For quantitative outcomes, basic statistics related to feasibility were gathered (eg, description of key recruitment numbers and adherence to CareFit from our in-app data, which included summaries across the Activity, Planner, Resources, and Sharing elements with time stamps of the time and date of use). Indicators of usability were underpinned by the SUS total scores, and on occasion, individual questions were used to support the interpretation of qualitative data. For all outcomes, differences in measures between baseline and follow-up were analyzed primarily regarding metrics that supported our understanding of feasibility and suitability of outcomes, such as completeness of the information.

## Results

### Description of Feasibility Study Populations

Our baseline demographics data describe 41 participants recruited (Tables 2 and 3). Most carer participants (39/41, 95%) were female, and all participants (41/41, 100%) self-identified as being White. A total of 63% (26/41) were educated to a degree level, and age groups varied from 25 to 34 years up to 75 to 84 years. Almost half (18/41, 44%) of carer participants had been caring between 3 and 10 years. Almost a third (13/41, 32%) reported caring for ≥8 hours per day, and 59% (19/32) of the council areas were represented. At baseline, the general adiposity (measured through BMI) varied from healthy (13/41, 32%) to morbidly obese (4/41, 10%). Activity levels as per the IPAQ-SF scoring were relatively equal across the vigorous (12/41, 29%), moderate (15/41, 37%), and low (14/41, 34%) groups. Muscular activity guidelines (≥2 days) were met by 39% (16/41) of the participants at baseline.

**Table 2:** Carer sample recruited at baseline.

			Participants, n (%)
**Gender (n=41)**			
	Woman or female (including transgender woman)	39 (95)	
	Man or male (including transgender man)	1 (2)	
	Preferred not to say	1 (2)	
	Described in another way	0 (0)	
**Ethnicity (n=41)**			
	Asian or Asian British	0 (0)	
	Black, African, Caribbean, or Black British	0 (0)	
	Mixed or multiple ethnic groups	0 (0)	
	White	41 (100)	
	Other ethnic group	0 (0)	
**Educational level (n=41)**			
	Degree or equivalent	26 (63)	
	Higher education	9 (22)	
	SVQ^a^	4 (10)	
	Other qualifications	2 (5)	
	School qualifications	0 (0)	
	No qualifications	0 (0)	
**Age group (years; n=41)**			
	18-24	0 (0)	
	25-34	2 (5)	
	35-44	4 (10)	
	45-54	9 (22)	
	55-64	14 (34)	
	65-74	10 (24)	
	75-84	2 (5)	
	≥85	0 (0)	
**Length of time caregiving (years; n=41)**			
	≤1	0 (0)	
	Up to 2	6 (15)	
	Up to 3	15 (37)	
	Up to 10	18 (44)	
	≥10	2 (5)	
**Time caregiving per day (hours; n=40)**			
	Up to 4	19 (48)	
	Up to 6	5 (12)	
	Up to 8	3 (8)	
	≥8	13 (32)	
**Mobile device (n=41)**			
	Apple	22 (54)	
	Android	19 (46)	
	Other	0 (0)	
**Location (n=41)**			
	Angus	5 (12)	
	Glasgow City	5 (12)	
	Highland	4 (10)	
	Dumfries and Galloway	3 (7)	
	Falkirk	3 (7)	
	North Lanarkshire	3 (7)	
	Perth and Kinross	2 (5)	
	East Renfrewshire	2 (5)	
	Midlothian	2 (5)	
	Moray	2 (5)	
	South Lanarkshire	2 (5)	
	Aberdeen City	1 (2)	
	Aberdeenshire	1 (2)	
	Clackmannanshire	1 (2)	
	East Ayrshire	1 (2)	
	Scottish Borders	1 (2)	
	South Ayrshire	1 (2)	
	Stirling	1 (2)	
	West Dunbartonshire	1 (2)	
**Study recruitment channel (n=41)**			
	JDR^b^	17 (41)	
	Other	7 (17)	
	Carers Scotland	6 (15)	
	Social media	4 (10)	
	Alzheimer Scotland or Brain Health Scotland	4 (10)	
	Health and social care	2 (5)	
	Age Scotland newsletter	1 (2)	
	Health and Social Care Alliance Scotland	0 (0)	
	Pharmacy advertisement	0 (0)	
**Study advertisement method (n=40)**			
	Email from research team	23 (58)	
	Social media posts	14 (35)	
	Conversations with health and social care professionals	2 (5)	
	Paper flyers	1 (2)	
**Approximate number of apps installed (n=40)**			
	>20	22 (55)	
	11-20	7 (18)	
	6-10	5 (13)	
	1-5	4 (10)	
	None	2 (5)	

^a^SVQ: Scottish Vocational Qualification.

^b^JDR: Join Dementia Research.

**Table 3:** Carer baseline health and activity data (N=41).

		Values
**BMI, n (%)**		
	Healthy	13 (32)
	Overweight	12 (29)
	Obese	12 (29)
	Morbidly obese	4 (10)
	Underweight	0 (0)
**IPAQ-SF^a^ score, n (%)**		
	Low	14 (34)
	Moderate	15 (37)
	Vigorous	12 (29)
**Sedentary behaviors (n=37)**		
	Proportion of the day (hours), mean (SD; range)	5.9 (2.4; 2-10.5)
**Sedentary breaks per day, n (%)**		
	>20	1 (2)
	11-20	3 (7)
	6-10	15 (37)
	1-5	12 (29)
	Did not know	10 (24)
**Muscular activity days, n (%)**		
	0	4 (10)
	1	12 (29)
	2	7 (17)
	3	4 (10)
	4	3 (7)
	5	2 (5)
	6	0 (0)
	7	0 (0)
	No answer	9 (22)
**Familiarity with activity guidelines, n (%)**		
	Had not heard of them	5 (12)
	Had heard of but were very unfamiliar with them	10 (24)
	Had heard of but were mainly unfamiliar with them	13 (32)
	Broadly aware of them	10 (24)
	Very aware of them	3 (7)
**Awareness of the benefits of physical activity, n (%)**		
	Not aware of them	0 (0)
	Aware of but very unfamiliar with them	1 (2)
	Aware of but mainly unfamiliar with them	4 (10)
	Broadly aware of them	20 (49)
	Very aware of them	16 (39)
**Current physical activity levels, n (%)**		
	Not active and no intent to start in the next 8 weeks	0 (0)
	Not regularly active but intent to start in the next 8 weeks	10 (24)
	Some activity but below the guidelines	17 (41)
	Regularly active, started <8 weeks before the study	1 (2)
	Regularly active, started >8 weeks before the study	13 (32)
**Muscular activity min in the previous week, n (%)**		
	None	6 (15)
	<10	7 (17)
	10-60	11 (27)
	61-120	3 (7)
	121-180	0 (0)
	181-240	0 (0)
	>240	2 (5)
	No answer	11 (27)

^a^IPAQ-SF: International Physical Activity Questionnaire–Short Form.

A total of 27 professionals participated in the interviews or focus groups, and 25 (93%) completed the detailed follow-up survey on the feasibility and implementation potential of CareFit. This population included pharmacy staff, those working in public sector roles across health and social care, charities and the third sector (at the national and local levels), public health roles, and digital health professionals, as well as those working in political roles. We do not detail each position individually to retain the anonymity of the participants (Table 4).

**Table 4:** Description of the professional sample.

Self-reported role	Experience (years)
Member of the Scottish Parliament	16-20
Health and social care partnership	6-10
Carer lead (health and social care partnership)	0-5
Senior role (Public Health Scotland)	11-15
Digital assistant director (health and social care charity)	0-5
Charity chief executive officer	6-10
Senior manager, social work	21-25
Leadership role (Digital Health & Care Innovation Centre)	0-5
IT director	0-5
Public health improvement practitioner	0-5
Professional advisor	6-10
Public health program manager	11-15
Pharmacist manager	0-5
Pharmacist	0-5
Pharmacist	0-5
Community engagement coordinator	0-5
Comanager (local carer center)	11-15
Nurse consultant for telehealth and telecare	11-15
Program manager	0-5
Technology-enabled care implementation officer	0-5
Planning manager	0-5
Trainee pharmacist	0-5
Medical doctor	≥26
Local area coordinator with a health and social care partnership	6-10
Principal officer	11-15
National Health Service worker	—^a^
Charity role	—

^a^Not available.

### Feasibility of Recruitment

#### Overview

We contacted 659 volunteers from JDR. In total, 88 carers of people with dementia (including n=34, 39% from JDR) showed an initial interest in the study, and 41 (47%) of them progressed to completing the baseline survey. A total of 41% (17/41) of our total study population was recruited from JDR. For the 24 carers who were recruited through direct advertisements, recruitment took place most notably through Carers Scotland, social media, and Alzheimer Scotland or Brain Health Scotland. The most successful nonregistry recruitment approaches were emails from the research team (23/40, 58%) and social media posts (14/40, 35%). Table 2 provides further information.

Qualitative data further improved our understanding through the identification of the 5 key subthemes outlined in the following sections. Table 5 provides an overview of the subthemes and further quotes.

**Table 5:** Minor themes under the major theme of feasibility of recruitment.

Themes	Example quotes
Lack of carer self-identification and related terminologies	“And for us in Glasgow ethnic minorities are actually quite a hefty proportion of our population, so non-English speakers, they’re really underrepresented at carer centres already, so they’re a big worry for us in terms of a community that we don’t feel are getting the right support even to begin with. And so I think at least having it in scope for the medium term to develop some translations. Or more accessible kind of mobile app functionality would be really helpful, yeah.” [Professional participant 12]
Digital readiness of informal carers of people with dementia	“...obviously a large number of carers are under particularly heavy stress loads and they’re getting bombarded with information all the time. And I think another app, another solution, quite often people just don’t pay attention to it because they’ve got enough on their plate as it is.” [Professional participant 6] “I think it’s like you say after you see them, maybe a couple of times, you understand this person’s probably confident enough with their phone or tablet. They could probably work to it, whereas someone who comes and asks you to help set up their e-mail and has other problems with technology at home.” [Professional participant 25]
Catering to different stages of the caring journey	“Because if a carer is at that point where their stress load is so high. They can’t take on doing something else then it it’s not really gonna be a benefit to them. But if you can get it in front of them and there’s probably quite a narrow window actually. When that is, when they’ve got, they’ve got the kind of headspace and the capacity to do it.” [Professional participant 6]
Increasing breadth and depth of advertising to identify informal carers	“I think it has to be much more direct, either telephone or face to face and finding the way to create like the right language to discuss these different things cause it’s a very diverse community. So a leaflet’s not gonna suit everybody and sometimes there’s a low level of literacy across some patients. So it has to be quite direct.” [Professional participant 14]
Coordination with key stakeholders, including using established “watering holes”	“You need to understand the habits of the people that you’re trying to reach. What sometimes people talk about it in terms of watering holes, so where do they go? Where do those other carers go? Do they have support groups?” [Professional participant 8]

#### Lack of Carer Self-Identification and Related Terminologies

A key challenge for recruitment was that people who care for others do not always identify with terminologies such as *carer* or *caregiver*. Such challenges are extended further across more demographic-specific barriers to research and access to health care related to gender or cultural background, personal viewpoints, or cultural beliefs:

The trouble, I suppose, is that you know, with carers, a lot of carers don't badge themselves in that way.

Professional participant 5

#### Digital Readiness of Informal Carers of People With Dementia

Stakeholders typically assumed carers of people with dementia to be older, less familiar with technology, and difficult to engage due to caring responsibilities.

The level of responsibility placed upon carers of people with dementia was highlighted by a number of professional stakeholders. The diversity in technology confidence and support requirements was highlighted across stakeholder interviews:

One of my questions might be is from the Alzheimer’s carers’ audience, they might be the ones that are in most need, but they might also be the hardest ones to actually get.

Professional participant 8

#### Catering to Different Stages of the Caring Journey

Targeting earlier provision in the journey of a carer was a common suggestion, ensuring that the intervention becomes part of postdiagnostic support. This could include linking with general practices, health centers, and carer centers. The consensus was that, as dementia progresses, caring needs placed on the carer will become too demanding to allow CareFit to be as effective or easy to implement. Linking with other services and organizations would be effective at the local level, but wider integration could be achieved if CareFit was able to align with national and regional policies, strategies, and agendas.

#### Increasing Breadth and Depth of Advertising to Identify Informal Carers

Suggestions from stakeholders regarding advertising varied considerably and included advertising approaches both in person and at distance, such as the use of local radio advertisements, social media, visibility on both the Apple App Store and Google Play Store, telephone contact, leaflets, and posters as well as more unstructured approaches such as word of mouth. In terms of the content of such materials, stakeholders mentioned that the benefits of using CareFit could be important to highlight upfront.

#### Coordination With Key Stakeholders, Including Using Established “Watering Holes”

Many stakeholders highlighted the need to coordinate at a local level with other services that carers already frequent, including “less formal” networks that may involve third sector presence and established local services. More broadly, stakeholders highlighted that face-to-face roles remain important for app uptake and use, where roles such allied health professionals or the pharmacy sector may complement future implementation. To a lesser extent, stakeholders also had some suggestions for coordination around national-level and local-level initiatives.

### Feasibility (Safety and Usability) of the Intervention

We first examined the feasibility of the intervention through the lens of safety—there were no adverse events reported by any of the participants involved in the study. In terms of usability, SUS responses were completed by 44% (18/41) of the participants. The average score was “acceptable” [36]. On the basis of the responses to the individual questions, participants were primarily positive toward the CareFit app (eg, 8/18, 44% strongly agreed that they would like to use CareFit frequently [question 1], and 15/18, 83% disagreed or strongly disagreed that CareFit required learning a lot of things to use it [question 10]).

### Adherence and Practical Considerations for Use: Informal Carer Data

#### Overview

A total of 41 participants initially joined the study, 38 (93%) downloaded the app, and 33 (80%) used it throughout the 8 weeks. In total, 51% (21/41) of the participants submitted the follow-up surveys (see the Methods section for further details). We explored use through 8 weeks of content management system data. Our overall “tag” counts (from 2891 instances) identified that the most prominently used parts of CareFit were the Learn tab (1296/2891, 44.8%; 1296 events), followed by the Activities (966/2891, 33.4%) and Planner (230/2891, 8%) tabs. Other available tabs were used to a lesser extent, such as Own activity (163/2891, 5.6%), Sharing (159/2891, 5.5%), and Resources (47/2891, 1.6%).

**Figure 3:**
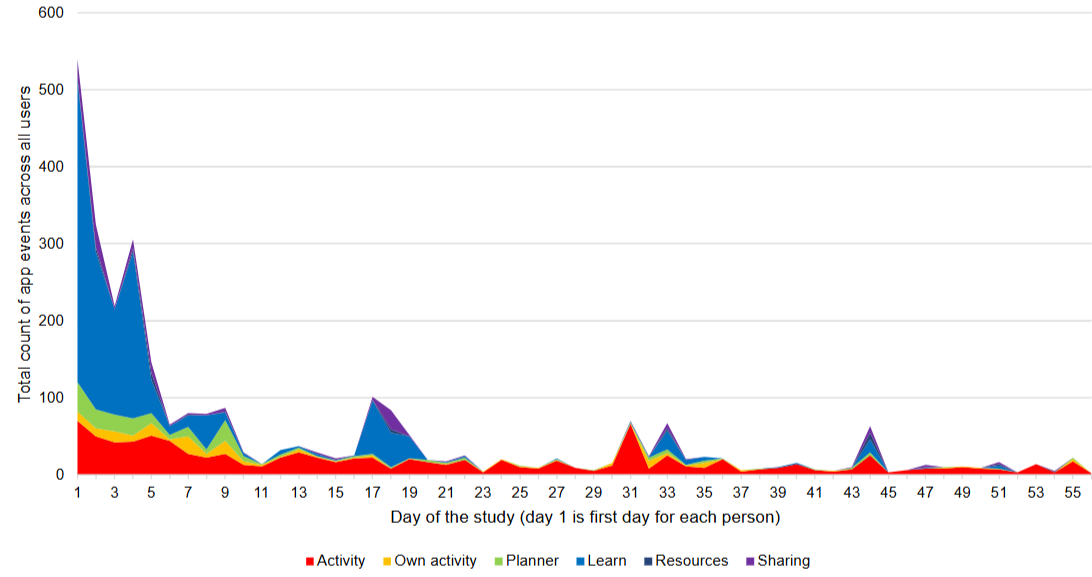
Overview of CareFit use by participants across the 8 weeks of the study relative to each user. This figure shows the total count across all users for different elements of the app, such as Activities, Own activities, Planner, Learn, Resources, and Sharing.

**Figure 4:**
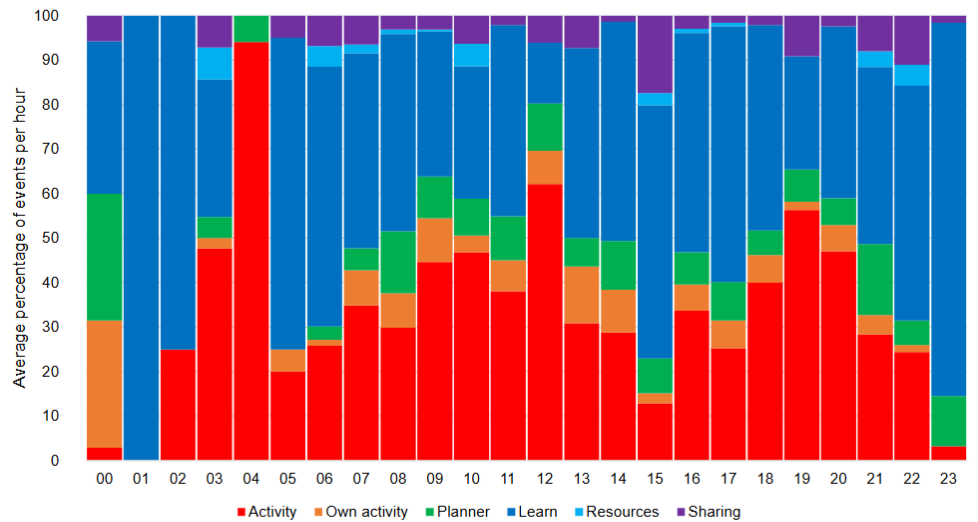
Overview of CareFit use in terms of the time of day across all users. This figure shows the total count across all users for different elements of the app, such as Activities, Own activities, Planner, Learn, Resources, and Sharing. It should be noted that use during the night was very low.

Events and associated time stamps (Figure 3) identified a large initial engagement with CareFit, particularly with the Learn tab (eg, week 1), as well as a subset of users who established steady engagement with the Activity section throughout the 8-week study. Small peaks of app use could be observed at the 3- and 5-week points. This timing coincided with 2 keep in touch emails sent directly to users on the platform to offer technical and then more general study support. Figure 4 shows use across different times of the day, where we observed higher use during preoffice hours, a reduction in use in the middle of the day, and late-afternoon to late-evening use.

We further explored adherence and practical considerations through themes generated from carer data (21 questionnaire responses and 8 qualitative interviews). We summarize these data through 5 key subthemes in the following sections (Table 6).

**Table 6:** Minor themes under the major theme of feasibility (and usability) of the intervention from informal carer data.

Themes	Example quotes
Caring routines	“...it’s not a kind of 24 hour seven day a week thing, you know, she’s not in the house with me, but it certainly impacts on any kind of routine that you would have had previously. So it really does help in in establishing a kind of routine and I suppose that reminder that you need to look after yourself if you’re going to be able to look after somebody else.” [Carer participant 7]
Motivation for physical activity	“I think it would be really useful for people like myself who are stuck at home. Who can’t get to a gym or even can’t go a walk. When I go a walk, It’s not far, because I can’t really go away and leave my husband. He’s not able to be left. So I find it it’s a good idea, it just maybe needs a bit of tweaking.” [Carer participant 1] “My well-being is really important and there are some days where I’m very very tired and you don’t always feel you have the energy to do the exercises.” [Carer participant 3] “Essentially that was what it did for me. It gave me motivation, even if it was only for a minute. And I felt quite positive at the time. And then other times I would look at it and go oh, can’t be bothered. But that’s my own. You know, that’s just me.” [Carer participant 1] “Other carers or other people who’ve been through the same thing. Not quite giving permission. But you know saying, this is good. This will...help you. This will make you feel better and that you are important and because you spend well for me as a carer you spend so much of your time advocating for the person you’re looking after and you know fighting services to get something in place. It’s quite exhausting. It’s actually being allowed to say you need time too.” [Carer participant 7]
Accessibility and usability of CareFit	“Part of that was because it’s on my phone, so it’s working via Wi-Fi. And our Wi-Fi goes down quite a bit here.” [Carer participant 1] “I did find the planner a little bit frustrating at times as it didn’t show the exercise I had added or had undertaken straight away. I did contact your team regarding this and you explained the reason behind that.” [Carer participant 4]
Content engagement	“Don’t wanna sound ignorant like I know what I need to do. I just need to actually do it. Like, yeah, so the whole purpose of the app for me was really more about getting me doing stuff rather than learning about what I needed to do.” [Carer participant 6] “I really enjoyed how I could see exactly what I’ve been doing and measure time wise. I’m quite a competitive person anyways, yeah. Quite enjoyed that element of seeing if I could do better than the last time.” [Carer participant 2] “I liked the videos because they were short and you could just get on and do like a very short burst of something and it felt manageable and it felt like he could achieve something rather than signing up to a 30 minute exercise class which you just were dreading.” [Carer participant 6]
Suggestions of improvements to CareFit for future use	“I think I would have preferred the videos to be a little bit larger as I sometimes struggle to see them on my phone and I suppose if I could have downloaded them onto my laptop then that would have been ideal for me.” [Carer participant 3]

#### Caring Routines

Regardless of the physical activity level achieved during the study period, most carers highlighted that accessibility to physical activity was associated with the demands of the caring role and being able to partake in activities “when you want.” Initiating physical activity around busy routines was something mentioned as a specific challenge to carers, where changes were supported through an appreciation of positive impacts, including those in the short term:

No, I enjoyed it. I didn’t use it probably as much as I should have, but that’s just because of things going on in my life.

Carer participant 1

#### Motivation for Physical Activity

Motivation for physical activity was an important point raised by many carers. While many carers felt that they knew why practicing physical activity would be important, both the impact and uncertainty of the caring role limited the opportunity for carers to practice regular physical activity. Other carers also commented on barriers to motivation for physical activity, such as feeling “exhausted” or environmental challenges such as poor weather. A number of carers mentioned that the app had helped change their perceptions of physical activity. Study adherence and practical considerations were explored through interviews and follow-up surveys:

I really enjoyed [using CareFit]. I thought it was easy to use and it fitted round about my caring duties. I found it very motivational at times and it made me think about my health. My physical health which I’ve ignored.

Carer participant 4

#### Accessibility and Usability of CareFit

Accessibility and usability of digital devices was a prominent topic in carer feedback. For example, first and early use of CareFit involved a number of steps to follow directed by the research team. Among 18 carer replies, installation was described as “easy” or “fine” by 15 (83%) participants, with 3 (17%) noting some issues or need for technical support:

[You] made it really simple. I work in a in a fairly similar role so I understand what the need for satisfying ethics and making sure that everything’s done so, no, I thought you did it very well actually.

Carer participant 2

Regarding longer-term use of CareFit, systemic challenges regarding technology (eg, mobile signal) were raised by participants, as well as more specific challenges regarding the design of CareFit.

Over the course of 8 weeks of use, a number of carers highlighted specific technical challenges regarding the use of CareFit. One challenge highlighted by a number of participants was the size of the physical activity videos on a phone screen, particularly when viewed at a distance. This included the need to maximize video size by putting the phone in a landscape orientation:

...the size of the screen that they offer for some of them. You know, and put your phone this way, put your phone that way. And it’s like when my phones way over there and I can’t quite see it.

Carer participant 7

In the earliest stages of the study, there were also specific technical issues with the activity planning and recording section of the app that a number of participants commented on. We also received more broad feedback on accessibility, including potential future challenges such as those regarding costs, where the app being free at the point of use would be particularly attractive.

#### Content Engagement

When asked which feature or part of the CareFit app was most useful, the survey responses received highlighted the activity videos (13/18, 72%). Other responses mentioned the activity planner (3/18, 17%), Community tab (1/18, 6%), and Learn tab (1/18, 6%). Notifications were suggested to hold utility in reminding participants to use the app, particularly in the earliest stages of use, but were also associated with pressure. The activity planning and recording was highlighted as a key strength by a number of participants. There was a range of comments regarding the Learn tab and associated content. Some carers found such information beneficial, but others pointed out that they felt that they already knew enough information to start on a physical activity plan before first use.

#### Suggestions of Improvements to CareFit for Future Use

A number of participants suggested ways to improve CareFit going forward. As mentioned previously, this included technical improvements suggested for the activity planner. Feedback included a focus on integrating additional devices and additional physical activities, increasing community elements, and improving aspects regarding the reminder notifications. For example, the use of a laptop would allow for a better screen size for participants to use.

### Adherence and Practical Considerations for Use: Professional Data

#### Overview

Study adherence and practical considerations were explored through interviews and follow-up surveys with professionals who worked with dementia carers. The 5 key subthemes identified are listed in the following sections (Table 7).

**Table 7:** Minor themes under the major theme of feasibility (and usability) of the intervention from professional data.

Themes	Example quotes
Addressing different levels of the digital divide	“I think in this modern day and age, I think that we can’t really hide under that stone anymore. I think is that if 2020 taught us nothing else, it’s that people actually can, when necessary, embrace technology and that is the sort...The reality is the sort of people who are going to use this, they’re going to be some people are into apps that that’s your target group, you know, it’s not the...some person is gonna ask if you have the video on VHS.” [Professional participant 26]
Timing and target group for intervention	“One of the biggest things that I hear from carer organisations is carers are the last people that will look after themselves and perhaps I don’t know what some of the statistics around carers are. But you know it’s beyond a full-time job. And it’s getting people to rethink their pre-existing habits I suppose.” [Professional participant 16]
Integration with other services	“Charities with access to carers and used by carers as a trustworthy source of information and already have an established relationship.” [Professional participant 23]
The need to embrace human elements and increase social connections	“I suppose it’s just, the health professionals, the ones whose health is their bag and wellbeing’s their bag to actively promote it as much as they possibly can. And thinking again people who go into people’s houses so, [Occupational Therapists] and staff, home care staff and things like that. Just making sure that they’re aware of it and just bring it into conversation. If ‘you used your app today? I will have the next half hour, why don’t you go and have shot at it?’” [Professional participant 3] “Charities with access to carers and used by carers as a trustworthy source of information and already have an established relationship.” [Professional participant 23] “So I’ve got one of those with things, the smart watches. And also like takes your heart rate and all that kind of stuff and when you go into the app, there is...There’s simply just here’s your reading. But click here for more information. It’s not busying the page, but it’s gonna say if you want, here you go, off you go and go and find that information...Delivery of information, don’t muddy it up too much.” [Professional participant 16]
Facilitators to support implementation and longer-term use	“The commissioners are the ones that you typically you would need to convince.” [Professional participant 8] “...that’s the that’s the trick with exercising that, like, you find something that’s fun, you know, and it ends up being good for you as a byproduct, you know. So if you can make it engaging then, more the more the better.” [Professional participant 16]

#### Addressing Different Levels of the Digital Divide

Stakeholders highlighted that the initial adoption of CareFit requires overcoming numerous barriers, including different levels of the digital divide. For example, dependency on having suitable technology and means to access the app can empower some and limit others. Connecting through Wi-Fi and mobile data or potentially needing to pay for a regular subscription to access CareFit may not be viable if carers’ finances are limited. Such barriers also extend to human and wider cost elements:

...it gets down to you actually have to practically show people how to use things. So you know apps being solutions on their own I think is never the answer. You need to have human interaction to show people the benefits of these things...

Professional participant 6

Digital literacy and skill were noted as potential barriers, especially as carers are typically assumed to be older and any new technology requires time to build familiarity and skill, among existing burdens. The data privacy and quality of the app would need to be guaranteed as well. There was also the recognition that some carers will simply not wish to engage with digital interventions and prefer traditional offline alternatives. At the same time, stakeholders felt that the prevalence of technology and recent experience of providing services online throughout the COVID-19 pandemic demonstrated that these concerns can be overcome in this population. Creating an offline version of CareFit reassured some concerns.

Digital divide concepts also extended to existing routines and practices. For example, carers may use other digital resources whether they choose to or out of necessity, but others may see them as a further burden on them (ie, another account and another password to remember). Digital literacy and language barriers may also prevent carers from accessing the support that CareFit can provide, and the app would require an accessible and usable interface to be effective (eg, minimal use of text, with interactivity and images being more important). Given the limited time and opportunity that carers have for physical activity, streamlining the process of using the app to complete intended tasks should be prioritized (eg, minimal steps to starting an activity).

#### Timing and Target Group for Intervention

Stakeholders were cognizant that creating new habits in carers requires careful consideration of the transient nature of caring and that presentation of CareFit at an optimal upstream timing was critical:

I see the caring journey in stages cause we try to adapt our thinking not to what we do, but what they do. So many organisations really get involved with carers when the caring is really critical and impactful, at which point are very unlikely to have time to think about the app, so you would want to go to the downstream, the green where the early intervention and getting things in there.

Professional participant 7

A critical barrier to achieving regular physical activity remained regarding habit and the ability to change the societal norm that carers are the last people who will look after themselves given their existing pressures and commitments.

The expectation placed upon carers that they should be exercising and using interventions such as CareFit has the potential for harm or burden, with stakeholders suggesting that carers could experience a sense of guilt if they could not effectively do so among other caring responsibilities. Guilt may also arise from the idea of taking time for themselves rather than focusing on caring despite the possible benefits that self-care can offer for both the carer and the person cared for. Carers are limited in available time and resources, especially as the condition progresses. They may also simply value other ways to use their downtime instead of exercising or practicing physical activity. Culture and language differences also play an important role in physical activity engagement.

Facilitators of habit formation included both designing an app that accommodates rapidly changing situations due to events such as hospital admissions; illness of the carer or the person cared for; and carers’ anticipatory grief, especially in later stages of dementia. All carers may face some limitations regarding opportunities for physical activity, and between individuals, the rate of behavior change, personal goals and end points, and activity levels can vary, requiring an adaptive approach to enable habit formation. Equally, the caring role can also change when the carer transitions from their role (eg, the person cared for passes on or the responsibility of caring is taken on by someone else) or if the carer reaches a stage where they no longer depend on CareFit to continue their new physical activity behaviors.

#### Integration With Other Services

Stakeholders stressed that CareFit should not be delivered in isolation but, instead, should leverage value and visibility from a wide variety of existing services at local, regional, and national levels to integrate into a suite of existing tools. However, similarly to many novel interventions and services, there remains a risk that the promotion and use of CareFit will become another thing to do that may not be practical given current workloads and roles:

If you’re able to bake that into what they’re already doing. Then you’re kind of winning on it.

Professional participant 16

Suggestions for how such an intervention could be both introduced and maintained included a number of professional roles and services. For example, this could include support from professional staff on house visits where informal carers would be present.

Feedback on integration included established digital health resources and tools from both NHS providers and dementia or caring charities. An example was local council physical activity websites or health and social care mobile apps (eg, the Jointly app developed by Carers UK).

Suggestions regarding integration also extended to wider digital commercial sectors and “consumer” marketplaces. Given that the market for physical activity and digital is already busy, connections to platforms such as Apple Watch and Apple Fitness+, Fitbit, and Strava can make interventions such as CareFit more attractive to users, especially those already using these different physical activity tools.

#### The Need to Embrace Human Elements and Increase Social Connections

Alongside suggestions of integrating the app into services and staff roles was also a broader need to embrace human elements across the trajectory of care. The persistence of the app across the carers’ local communities and environments was a strategy proposed to secure engagement through demonstrations, testimonials from “champions,” and presence at regular activities and events within the community (eg, Digi-PALS library technological support, coffee mornings, or social events). At the onboarding level, this included critical conversations that professional stakeholders such as pharmacists have with carers, including “chatting them through what care fit is and how it could benefit them.” Motivators for carers to adopt CareFit included carers championing their own use, stories with case studies, and testimonials highlighting the benefits and minimal demand on time and energy required to use CareFit. The potential value of CareFit could also be expanded to directly reduce isolation, possibly through participation with other carers in group activities and social features or even as a topic of discussion within the community. Linking to existing platforms, including those outside of the health and social care settings (eg, social media, Alexa, and Strava) could also encourage greater adoption by carers:

So you know and maybe monitor your CareFit progress or how you’re using it, how’re you going and you know make it more of a kind of social element to it. So I suppose that’s a lot of people, you know, like that team type thing with their friends and stuff like that. So any opportunity to maybe kind of you know make it with a kind of social thing would help at all.

Professional participant 3

#### Facilitators to Support Implementation and Longer-Term Use

Facilitators to support implementation and longer-term use included suggestions at both the professional organization level and the individual informal carer level. At the organizational level, addressing the financial and structural models was raised by a number of stakeholders. This included generating knowledge of the organizational infrastructure to establish which key decision makers could initiate use of CareFit even on a small scale. The cost, resources, and effort to maintain such an intervention were also raised by stakeholders, more so if they aimed to provide technology and means to access the intervention as well as training and support.

Organizations may also be hesitant to implement new technology, with the development, provision, and maintenance of digital interventions imposing financial costs and more demand on already burdened staff who, similarly to carers, may be opposed to the introduction of new technologies and approaches. A strong evidence base and guaranteed uptake with users is required to assure organizations that there is minimal risk in implementing the proposed technology and that existing inequalities will be addressed rather than exacerbated (eg, having to provide access and connectivity to use an intervention for those who may not have the means).

Professional stakeholders largely appreciated that longer-term delivery of CareFit would require financial sustainability, although there was not a consensus on how this could be achieved:

As decision-makers and commissioners as well as policy-makers, if an intervention had clear benefit it might be able to benefit from some kind of national funding. But more likely that we would expect local providers to fund from existing budgets.

Professional participant 11

I think the payor is most likely to be the public sector but ideally CareFit is being delivered as a business solution or service to that market.

Professional participant 8

At the individual carer level, several suggestions were made to support the use of CareFit in the longer term, including beyond the initial 8-week study outlined in this paper. First, CareFit must be accessible and available to carers, whether this is through incorporating it into a suite of existing tools and resources or advertising through leaflets and posters with QR codes. For specific devices, CareFit should be available through app stores and use native functionality and services to ensure that the app is passive and requires minimal effort to start using it. Updates and new content would also be required to keep interest in the app but should not compromise familiarity or fundamentally change the CareFit app, which may discourage users instead. This would ideally make carers’ feedback result in effective changes and improvements and keep information and content up-to-date and relevant.

Prompts, reminders, and notifications from the app itself could be important tools to ensure consistent engagement throughout long periods but should be designed to be motivational and encourage the user to want to engage in physical activity rather than just reminding them of a need to complete another task. Gamification and rewards such as challenges and goals where a defined target needs to be reached can similarly encourage engagement. Rewarding continued engagement, such as streaks (eg, completing an activity each day or week), can also be essential to habit forming but can be dismaying when disengagement, which may not be possible for this user group to control or predict, is punished. Competing with other users, such as leaderboards or group progression toward a shared goal, were also suggested as engaging features suitable for some users.

Social connectivity was described in other features besides competing, such as social feeds or forums to share successes and receive support from peers on the app or using physical offline groups, activities, or classes to encourage use and check in on how CareFit is working for carers. These ideas were particularly supported as the caring role can be isolating and provide limited opportunities to connect with others. There were also suggestions to integrate physical activities that could be completed by both the carer and the person living with dementia (eg, chair-based exercises or joint walks), removing the need to find separate time and space.

### Feasibility and Suitability of Outcomes

#### Overview

We explored the feasibility and suitability of outcomes across outcome measure response data completeness and the subjective utility of measures through professional stakeholder interview feedback (including unintended consequences; Table 8). Outcome measures were broadly well completed at both baseline and follow-up (Table 9). Some scales such as the IPAQ-SF showed completeness for some but not all questions; for example, measures of walking activity and sedentary behavior had a >90% completion rate. However, IPAQ-SF vigorous and moderate activity measures were less well completed, with response rates of 81% (17/21) and 76% (16/21), respectively, at follow-up. It was not clear whether the omitted values were because individuals did not complete activities within a specific intensity category or because they missed the question altogether. We included a question on breaking up sedentary behavior as well as a question on muscle and balance (Multimedia Appendix 4) as these are included components of the current physical activity guidelines and were integrated into the CareFit app. Both our question on breaks from sedentary time and muscular activity (days) question were relatively well completed, with response rates of >75% at baseline and follow-up.

Professional stakeholders highlighted both short-term and utility metrics of interest, including those based on measuring usability, including from professional perspectives; direct improvements in physical activity levels and sedentary behavior; broader (secondary) impacts and unintended consequences; and inclusiveness of marginalized groups and those facing inequalities.

**Table 8:** Minor themes under the major theme of feasibility and suitability of outcomes.

Themes	Example quotes
Measuring usability, including from professional perspectives	“For example, disability organisations...Getting them to even test it and see how they can actually use it is it is it actually usable, you know? And we do they understand is it easy, easy to use? And does it tell them then in a way that they would then understand so, you know?” [Professional participant 24]
Inclusiveness of vulnerable groups and those facing inequalities	“And yeah, that someone might need that really intensive part to get them on their feet. But once they’ve got the tools etcetera to be active. This could really help them maintain that, so yeah.” [Professional participant 4] “But for me, one of the biggest factors of this is carer resilience, for us is a huge support in terms of preventing admission, supporting early discharge.” [Professional participant 26]
Measuring broader (secondary) impacts and unintended consequences	“We’ve been, ideally, from my perspective, sitting in public health, we would like to see a narrowing of health inequalities. So for me, I would like to see improved access to physical activity, improved access to mental health through the app, so that kind of sign posting or navigation to local service for me feels really important. And I think a consequence of the opposite stands at the minute might be a widening of inequalities because actually we’re helping the well-resourced, wealthy, perhaps or well educated.” [Professional participant 12]

**Table 9:** Completeness of data at baseline and follow-up.

Question group and question		Responses at baseline (n=41), n (%)	Responses at follow-up (n=21), n (%)
**IPAQ-SF^a^ and additional physical activity measures**			
	Vigorous activity	27 (66)	17 (81)
	Moderate activity	30 (73)	16 (76)
	Walking activity	40 (98)	19 (90)
	Sedentary behavior	37 (90)	20 (95)
	Breaks of sedentary behavior^b^	31 (76)	16 (76)
	Muscular activity (d)^b^	32 (78)	16 (76)
	Muscular activity (min)^b^	30 (73)	16 (76)
**Confidence and barriers to physical activity**			
	Tiredness	41 (100)	20 (95)
	Bad mood	41 (100)	20 (95)
	Not having the time	41 (100)	20 (95)
	Vacation	41 (100)	20 (95)
	Rain or snow	40 (98)	20 (95)
**Knowledge and awareness of physical activity benefits and guidelines**			
	Knowledge of physical activity guidelines	41 (100)	20 (95)
	Awareness of benefits of physical activity	41 (100)	20 (95)
	Stage of change	41 (100)	20 (95)
**EQ-5D-5L**			
	Mobility	41 (100)	20 (95)
	Self-care	41 (100)	20 (95)
	Usual activities	41 (100)	20 (95)
	Pain or discomfort	41 (100)	20 (95)
	Anxiety or depression	41 (100)	20 (95)
	Numerical scale of health	41 (100)	20 (95)
**SUS^c^**			
	Full scale (10 items)	N/A^d^	18 (86)

^a^IPAQ-SF: International Physical Activity Questionnaire–Short Form.

^b^Measures that were developed within this study as described in Multimedia Appendix 4.

^c^SUS: System Usability Scale.

^d^N/A: not applicable.

#### Measuring Usability, Including From Professional Perspectives

Initial assurances for uptake included usability, ease of use, and acceptability of CareFit. For example, early evidence for use could be obtained by facilitating continued feedback from users and testing with disability organizations or those that advocate for the needs of people with disabilities. Stakeholders were largely reassured of the quality of the project through the incorporation of co-design from the outset. They suggested that use cases and real experiences of users would be useful demonstrations of effectiveness and ease of use.

#### Measuring Broader (Secondary) Impacts, Including Unintended Consequences

In terms of measuring changes over time, stakeholders were interested in seeing increases in initial physical activity levels, particularly continued or maintained physical activity. While physical activity increases are recognizably positive, providing support for users to understand the outputs and results of CareFit is also necessary to assist them in sustaining such behavior in the long term. Delivering the right type and level of support to an individual is also important. This may involve linking with other services, such as more intensive referral programs that run for several months before users reach a stage in which tools such as CareFit become viable for more independent exercise and activity.

Other broader outcomes described included quality of life, well-being, number of crisis points (ie, unplanned admission of the carer or the person cared for into hospital settings), hopelessness, frailty, and mortality improvement measures. These measures may also provide helpful insights apart from measuring just physical activity and help provide a better representation of an individual’s progress. The topic of person-centered support and informed goals was also mentioned when discussing effectiveness, giving control of the goals and progress to the individual carer rather than applying formative assessments to the population as a whole. Stakeholders often discussed how individual carers presented with needs for different types of support (eg, health and well-being, legal, financial, and emotional) alongside their own goals. Addressing this with individual carers was often the approach of many stakeholders, with personal goals still linking with the goals of the wider service or organization. Empowerment of carers and the building of confidence and skills for effective self-management were encouraged:

So move to personal outcomes, a formative assessment, have people—what you’re asking people to invest in “what are you gonna do, not what we’re going to do for you.” What are you going to do for yourself? And then benchmark that, three months ago, or whatever time scale, a week ago. You said you would do this. How have you done this week?...But empower and enable people to be in control of that rather than do it to them.

Professional participant 7

From professional feedback, common unexpected consequences of CareFit that were discussed included increased carer resilience, more effective self-management, and more confident and able carers.

The subject of unintended consequences was additionally explored through carer feedback. While most carer data did not suggest any unexpected benefits, feedback included the person with dementia taking part in physical activity, completing physical activities but not noting the activity within the app, and becoming more competitive with themselves as carers.

#### Inclusiveness of Vulnerable Groups and Those Facing Inequalities

While promoting self-management and resilience may benefit the system supporting carers, concerns were raised that marginalized and vulnerable groups would continue to struggle even if CareFit were to be successfully implemented. Directly addressing a range of public health behaviors in carers through the use of CareFit would be valuable to sectors such as public health and for gaining support but would require long-term use and longitudinal data collection with thoroughly planned methods and measures. While dementia is a long-term condition and there is potential to support carers over these periods, unpredictable routines can make continued regular use hard to guarantee and enforced timescales less feasible.

## Discussion

### Principal Findings

We successfully co-designed, expanded, personalized, and evaluated the implementation potential of a mobile health app to support physical activity in carers of people with dementia. Throughout, our approach was to learn about the real-world practicalities regarding implementation guided by both the MRC complex intervention framework [24-26] and the RE-AIM framework [27]. Our co-design outputs built significantly on existing research through establishing a greater depth and improving the functionalities of CareFit. By building a bespoke content management system, we established a future capacity within the platform to capture real-time knowledge of carer well-being and deliver key messages to carers in local community settings, including at scale. Self-identification regarding being a carer appears critical for uptake—early introduction of CareFit, including through face-to-face interactions, appeared extremely useful. Intervention uncertainty findings highlight positive feedback on the graphical user interface; however, dropout of carers at 8 weeks was notably high at 49% (20/41; 21/41, 51% of the participants completed the study). Finally, while completeness of outcome measures was relatively good, there remained gaps regarding key validated measures such as the IPAQ-SF. We included 2 novel measures of sedentary breaks and muscle and balance activities, and these were largely well completed. These are important components of the current physical activity guidelines that remain particularly easy to overlook.

### Limitations

Despite a number of achievements, several limitations remain. Due to time constraints, we did not apply for UK Health Research Authority approval to recruit participants in receipt of NHS services. This additional recruitment channel may have increased reach and helped us achieve the target sample size, improved the specificity of recruitment of individuals with a formal diagnosis of dementia, and facilitated the wider engagement of professional roles such as physiotherapists and occupational therapists. In terms of suitability of outcomes, our reliance on self-reporting regarding physical activity levels permitted individuals to join our study who were already regularly practicing physical activity at higher levels than those desired for our target population and, thus, less likely to benefit from our intervention design. The research purpose of self-reported physical activity levels was as a baseline and follow-up measure, not as a “screening” question. While information sheets can always be improved, there was clear reference to physical activity inclusion criteria. Future research will ensure that eligibility criteria are revised and participants’ activity levels are considered more clearly ahead of them consenting to take part in a study. Finally, our use of RE-AIM was ultimately focused on the planning and conduct of data gathering as opposed to the evaluation phases. While flexibility regarding the use of RE-AIM is both permitted and encouraged, as a research collaboration, our focus from the outset of this work was to better understand uncertainty regarding recruitment, intervention design, and suitability of outcomes following elements of the MRC complex intervention framework. We chose to focus on these elements to provide a clearer narrative throughout.

### Comparison With Prior Work and Future Directions

A key finding was that carers of people with dementia were recruited at a slower-than-anticipated rate. In the context of a randomized controlled trial (a future consideration for this work), our recruitment rate of approximately 1 to 2 carers per week through 1 center was higher than medians reported elsewhere [37]. Nonetheless, we did not achieve our intended target of 50 carers of people with dementia, and future work should consider ways to mitigate the risk of slow recruitment, including increasing the length of the recruitment period (ie, beyond 6 months) or considering multisite recruitment over a larger geographical area and broader carer groups. The self-identification and visibility of all carers has been highlighted across a wide array of recent literature [38,39], and other recent work has also highlighted limited uptake of interventions for carers integrated with health care service delivery even in sizable population groups [40]. Future work should consider stronger emphasis on face-to-face conversations, which can be critical for uptake and retention [41]. Despite efforts to add more representation of carers (eg, gender, ethnicity, and geographical setting), inequity in the reach of CareFit was a prominent observation. This may be related to intersecting sources of inequity and disadvantage; for example, a lot of carer research demonstrates a high prevalence of individuals who are female, White, and highly educated [40]. Further qualitative work is required building on foundational knowledge [42-46] as to how we can design preventative approaches to reach those traditionally marginalized in a more equitable manner.

Arguably, one of the most pertinent findings regarding intervention uncertainty was a study dropout rate of 49% (20/41; 21/41, 51% of the participants completed the 8-week study). While this is high in comparison to clinical trials [47], there is systematic review evidence suggesting that high attrition can be common for app-based observational studies [48]. Our additional exploratory analysis of data identified that retention was considerably lower for those carers with high levels of physical activity as opposed to those with lower levels (retention of 5/14, 36% vs 16/27, 59%, respectively). Thus, future work needs to consider how to achieve stricter enforcement of inclusion criteria. CareFit could also be expanded to cater to more stages of change according to the transtheoretical model and signpost such users to other care providers and professionals where required. While our technical issues were few in number, such occurrences are likely highly impactful to carers—both we and others recognize the heterogeneity of motivations present in this population, where personalized delivery is key [7]. Future work would ideally leverage learnings on the digital divide that both we and others [49,50] have noted. For CareFit, this could include reducing the number of download steps for first use, improving the flow of the activity planner (including creating a menu of potential plans), and increasing opportunities to use larger screen sizes such as laptops.

More broadly, facilitators for use across carers and professional stakeholders include integration into the realities of the caring routine and sustaining motivations for physical activity within this context. Many participants highlighted that the app as delivered was fun and motivational and the delivery of short videos (eg, including content for use in 1 minute) in particular could fit into the caring role. While this type of delivery method is relatively novel, technical challenges remained, some relatively addressable (eg, optimization of the planner) but also some more substantial, including those regarding the phone screen size being too small to allow physical activities to be easily conducted. Participants also mentioned that deepening links between the Learn and Activity sections would support carers with content “more about getting me doing stuff.” Analyses also highlighted an appreciation of the fact that human elements are critical to convert autonomy and competency regarding physical activity in carers into long-term maintenance. This includes “championing” of CareFit by other carers—mirroring the importance of social influence across a broader range of behavior change interventions [51]. We hypothesize that future efforts could leverage value both from face-to-face settings (eg, working alongside employers) and from community pages already set up on the app to date, including facilitating more content generation from other carers on a regular (eg, weekly) basis. Stakeholder analysis identified that optimal delivery and support could include a variety of settings, including the use of regular carer assessments, linking more closely with frontline community staff such as pharmacists and those conducting home visits to support adoption and regular use.

Our third key area of interest was the suitability and utility of different outcome measures of the (future) impact of CareFit. Qualitative findings from professional stakeholders suggest no clear consensus on what or how to measure regarding impacts. We conclude that, from a care provider perspective, current economic and staffing pressures mean that cost-effectiveness and reducing demands in care is likely to be a key driver of uptake. Concerns that health is not valued as an outcome it its own right have been raised in other studies [52]. While increasing physical activity levels in marginalized groups could yield significant long-term benefits for individuals and care providers, the short-term impact is critical. Our findings highlight the value in aligning with carer-described goals—something that would align with multiple potential models of carer empowerment [53,54] and that would be of significant interest for future work.

Throughout the work presented in this paper, we relied largely on self-report measures. While the validity of measures such as the IPAQ-SF has been well demonstrated across many different population groups [34], a recent meta-analysis identified low criterion validity for single items, such as single-item sedentary behavior questions [55]. Further development of (single- or multiple-item) measures with greater validity could be of interest to a variety of stakeholders. This could include further exploration of the new outcome measures introduced in this study. With respect to CareFit, measures could take on a dual role as both mediators (advancing the theoretical understanding of CareFit) and creating evidence-based and personalized feedback for carers. Missingness regarding physical activity measures included both activity recording within the app and the use of the IPAQ-SF. A more systematic and focused approach on understanding the recording of physical activity among marginalized groups appears to be of value. We propose that a helpful extension to CareFit (at least for some users) would be to integrate more automated data collection, such as the use of wearables or step counts (eg, collected using mobile phones). Such integration would have to be considered carefully so that those carers with lower health literacy or subject to the digital divide are still able to receive the intervention equitably.

### Conclusions

Accessible physical activity for carers of people with dementia remains an unmet societal need. Our work demonstrates that self-recognition and reach of carers of people with dementia are critical. While we cannot currently recommend the progression of CareFit to a randomized controlled trial, both the safety and usability of CareFit were promising. Furthermore, our work builds new knowledge on how to design interventions for carers—particularly the use of extensive video content, which was positively received. We conclude that CareFit sits largely within the context of preventative medicine as many informal carers are facing additional barriers to physical activity compared to noncarers. Further work is warranted to build foundational knowledge on how to further enhance both reach and adherence across key public health behaviors for this critical societal group.

## Appendix

Multimedia Appendix 1 COREQ (Consolidated Criteria for Reporting Qualitative Research) checklist items.

Multimedia Appendix 2 Example semistructured interview and questionnaire for informal carers of people with dementia.

Multimedia Appendix 3 Example semistructured interview and questionnaire for professionals.

Multimedia Appendix 4 Novel question on sedentary breaks as well as muscle and balance activities.

## Abbreviations

COREQ: Consolidated Criteria for Reporting Qualitative Research
IPAQ-SF: International Physical Activity Questionnaire–Short Form
JDR: Join Dementia Research
MRC: Medical Research Council
NHS: National Health Service
RE-AIM: reach, effectiveness, adoption, implementation, and maintenance
SUS: System Usability Scale

## Data Availability

The datasets generated or analyzed during this study are available from the corresponding author on reasonable request.
