# Supplementary material for: A Mobile App (CareFit) Supporting Physical Activity for Informal Carers of People With Dementia: Mixed Methods Feasibility and Adaptation Study
Source: J Med Internet Res. 2025 Aug 29;27:e56739. doi: 10.2196/56739 (PMC12432464; doi:10.2196/56739)
Supplement: Multimedia Appendix 2 [file jmir_v27i1e56739_app2.docx]

**Supplementary Appendix A2** Example semi structured interview/questionnaire for Informal Carers of people with dementia

| **Reach** |
| --- |
| How can we reach more carers to improve physical activity levels?  How do you think we can best reach carers of people with dementia in particular? |
| **Effectiveness** |
| What are the key changes or outcomes [from CareFit] that would allow your organisation to implement this kind of work?  ***Prompt*** Organisational perspective, such as cost-effectiveness or reduced workload? |
| **Adoption** |
| What barriers or issues do you see to CareFit being adopted?  **Follow-up** How do you think we can overcome these?  Can you think of any carer-based motivations for adopting CareFit that would help us get more carers to the point of first use? |
| **Implementation** |
| How would you see CareFit optimally working in the future if it were to be implemented?  How do you see CareFit integrating into service delivery (alongside other digital/dementia focused interventions such as iSupport)? |
| **Maintenance** |
| What do you feel could be done to help improve carers’ ability to continue to use CareFit consistently over time? |
| **Other** |
| Is there anything else you would like to add? |
