# Supplementary material for: A Mobile App (CareFit) Supporting Physical Activity for Informal Carers of People With Dementia: Mixed Methods Feasibility and Adaptation Study
Source: J Med Internet Res. 2025 Aug 29;27:e56739. doi: 10.2196/56739 (PMC12432464; doi:10.2196/56739)
Supplement: Multimedia Appendix 3 [file jmir_v27i1e56739_app3.docx]

**Supplementary Appendix A3** Example semi structured interview/questionnaire for professionals

| Thematic areas | Example research question |
| --- | --- |
| Reach | - How does your organisation reach informal carers currently to help them look after their own health and well-being? - Within these, are there any specific strategies you know of that can support reaching more marginalised groups of informal carers? |
| Effectiveness | - What outcomes or changes for informal carers and their health and well-being does your organisation look for? - How would you capture unintended consequences? |
| Adoption | - How do you think your organisation could best utilise technology like CareFit? - Has your organisation tried to implement technology like CareFit before? If yes, how did the implementation of the technology go? |
| Implementation | - Has your organisation experienced any barriers or issues to implementing such interventions for carers? If yes, please explain. |
| Maintenance | - Can you imagine technology like CareFit becoming a routine part of your organisation’s practices? Why? - What elements of your existing organisational infrastructure do you think might support a technology like CareFit in the long term? - What long-term effects do you think technology like CareFit may have for your organisation as whole? |
