# Supplementary material for: A Mobile App (CareFit) Supporting Physical Activity for Informal Carers of People With Dementia: Mixed Methods Feasibility and Adaptation Study
Source: J Med Internet Res. 2025 Aug 29;27:e56739. doi: 10.2196/56739 (PMC12432464; doi:10.2196/56739)
Supplement: Multimedia Appendix 4 [file jmir_v27i1e56739_app4.docx]

**Supplementary Appendix A4** Novel question on sedentary breaks alongside muscle and balance activities

**Question on Sedentary behavior breaks:**

| How many times would you purposely break up your sitting time in a day? |
| --- |

**Question on muscle and balance activities**

| The physical activities you do have the potential to improve your **muscular strength.**These exercises would be enough to make your muscles feel some tension, shake or feel warm.   1. How many days in the last week have you done**muscular strength**exercises (e.g. heavy gardening, heavy household chores, carrying heavy shopping or any other muscular strength exercises)?   Sub question:  1a. Thinking about your answer above. What are your total minutes of muscular strength activity in the last week? |
| --- |
